# Implantable Ultrasound‐Powered MXene/PVA Hydrogel‐Based Generator for Treatment of Glioblastoma

**DOI:** 10.1002/advs.202309610

**Published:** 2024-12-12

**Authors:** Xiaoping Hu, Ziyi Qiu, Yilin Yang, Ting Xu, Kai Sheng, Weicheng Lu, Jingdun Xie, Bingzhe Xu

**Affiliations:** ^1^ School of Biomedical Engineering Sun Yat‐sen University No. 135, Xingang Xi Road Guangzhou 510275 P. R. China; ^2^ School of Biomedical Engineering Shenzhen Campus of Sun Yat‐sen University No.66, Gongchang Road, Guangming District Shenzhen 518107 P. R. China; ^3^ Faculty of Engineering Department of Electrical and Electronic Engineering The University of Hong Kong Pokfulam Hong Kong SAR 999077 P. R. China; ^4^ Department of Anesthesiology State Key Laboratory of Oncology in Southern China Collaborative Innovation for Cancer Medicine Sun Yat‐sen University Cancer Center Guangzhou Guangdong 510060 P. R. China

**Keywords:** glioblastoma, MXene/PVA hydrogel, TTF, ultrasound‐powered

## Abstract

Glioblastoma (GBM) is a lethal disease with a poor prognosis due to its strong infiltration, which makes it difficult to remove completely. In this study, an implantable, modulus‐tunable, and ultrasound‐powered MXene/PVA hydrogel‐based tumor treatment device (UP‐MPH‐TTD), which generates specific electromagnetic alternating fields that disrupt the mitosis of cancer cells without adversely affecting normal neurons is developed. The MXene/PVA hydrogel is used to form a tumor treatment field due to its high biocompatibility, excellent flexibility, and high conductivity, which improves ultrasonic electrical conversion efficiency and significantly reduces the size of the equipment. The implantable UP‐MPH‐TTD is wirelessly ultrasound‐powered, small‐sized, lightweight, and simply structured, significantly boosting therapeutic efficiency and reducing restrictions on patient movement. In vitro and in vivo experiments confirmed the device's therapeutic effect, demonstrating a ≈92% inhibition rate in the growth of clinical tumor cells and a 73% reduction in tumor area in tumor‐bearing mice. The promising results indicate the broad application potential of the device in the treatment and prognostic improvement of GBM.

## Graphical abstract

An implantable, ultrasound‐powered MXene/PVA hydrogel‐based device introduces a novel approach to glioblastoma treatment by generating targeted electromagnetic fields to disrupt cancer cell mitosis. Combining high biocompatibility, flexibility, and conductivity, this lightweight, wireless system enhances therapeutic efficiency while minimizing patient restrictions, offering a groundbreaking solution for glioblastoma management.

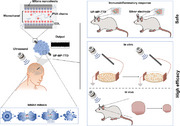

## 1 Introduction

Glioblastoma (GBM) is one of the most aggressive cancers that originate in the brain.^[^ ^1^ ^]^ Despite significant developments, the poor prognosis of GBM remains a serious clinical issue.^[^ ^2^, ^3^, ^4^ ^]^ Due to the pronounced infiltrative growth of GBMs, a cure through complete resection of the tumor is unfortunately not possible.^[^ ^5^, ^6^ ^]^ Systemic and locoregional treatments have traditionally been used and tested to improve prognosis.^[^ ^7^, ^8^, ^9^ ^]^ The average survival time is ≈15 months with traditional methods.^[^ ^6^, ^10^, ^11^, ^12^ ^]^ Recurrence and GBM resistance are the leading causes of patients’ death. Tumor treatment field (TTF) represents a promising new approach for GBM treatment. It is a physical therapy that uses alternating electric fields with low‐intensity (1–3 V cm^−1^), intermediate‐frequency (100–300 kHz) to inhibit cancer proliferation.^[^ ^13^, ^14^, ^15^ ^]^ TTF treatment has a lower toxicity profile than traditional treatments, with side effects limited to mild to moderate skin irritations.^[^ ^16^ ^]^ Promising results from TTF trials led to the approval of the first‐generation TTF device by the United States Food and Drug Administration (FDA) in 2011.^[^ ^15^, ^17^ ^]^ However, the traditional TTF system requires pretreatment of the patient's scalp and electrode routing on the scalp surface,^[^ ^18^, ^19^ ^]^ making it difficult to use and inefficient. Additionally, the low spatial resolution of scalp electrodes results in high power consumption and low treatment accuracy.

Wireless implantable devices offer a solution to overcome the limitations of traditional TTF, which eliminate the need for complex electrodes and routing. Additionally, implantable devices offer greater flexibility, enabling precise tumor treatment and reducing energy consumption. Currently, energy transmission and conversion are among the main challenges facing wireless implantable devices, and ultrasound holds great potential in biomedical applications.^[^ ^20^, ^21^, ^22^ ^]^ The propagation of ultrasound through a porous medium generates an electric signal, similar to the well‐known electroacoustic effect in the dispersions of mobile particles.^[^ ^23^ ^]^ This electroacoustic effect is known as the streaming vibration potential (SVP) effect. Moreover, the low propagation loss of ultrasound allows for greater penetration depth in the body and reduces unnecessary biohazards.^[^ ^24^, ^25^, ^26^ ^]^ Recent studies have shown that MXene and polyvinyl alcohol (PVA) can form a 3D network structure of hydrogel (M‐gel).^[^ ^22^, ^27^, ^28^, ^29^ ^]^ M‐gel exhibits significantly enhanced performance compared to MXenes or hydrogels alone due to the synergy created by the binding between the 2D structure MXenes with a negative surface charge.^[^ ^30^, ^31^ ^]^ These M‐gel generators can convert ultrasonic power into electrical energy based on the principle of SVP.^[^ ^22^, ^32^ ^]^

In this study, we designed a wireless implantable ultrasonic device based on MXene/PVA hydrogel (M‐gel). The device consists of an M‐gel core and a bio‐glass enclosure, with the entire system integrated into a small cylinder (radius ≈0.6 mm). Through in vivo and in vitro experiments, we demonstrated the device's remarkable efficacy in inhibiting glioma cell proliferation.

## 2 Results and Discussion

### 2.1 Working Mechanism and Characterization of UP‐MPH‐TTD

**Figure 1:**
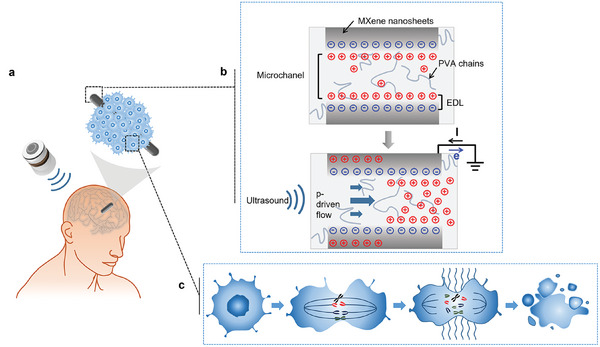
Schematic and operating principles of the UP‐MPH‐TTDs. a) Schematic diagram of an implantable UP‐MPH‐TTD for brain tumor treatment. b) Schematic showing the proposed mechanism for voltage generation phenomenon in the MXene/PVA Hydrogel. c) TTF inhibits brain tumor cell growth by disrupting cell division during mitosis.

The structure of the implantable, modulus‐tunable, ultrasound‐powered MXene/PVA hydrogel‐based tumor treatment device (UP‐MPH‐TTD) is shown in **Figure** **1**a. The main body of the device consists of a uniform columnar structure composed of M‐gel. The power generation principle of the M‐gel is illustrated in Figure 1b. When ultrasound is applied to the device, these microchannels in M‐gel are compressed, leading to a pressure‐driven flow (P‐driven flow) that drags counterions from the electrical double layer (EDL) outward. This movement results in a potential difference along the direction of ultrasound propagation between the exposed negatively charged surface and the cations accumulating in the flow. This potential difference induces additional electrons to move toward the external grounded electrode on the MXene nanosheet. When the ultrasonic pressure subsides, the compressed positive ion layer relaxes, causing electrons to flow back to the MXene layer. Consequently, as the ultrasound continues, the number of cations in the EDL within the MXene network oscillates, generating an alternating electrical signal. To facilitate the implantation of the device and prevent the expansion of hydrogel in water, borosilicate bioactive glass is used to encapsulate it.^[^ ^22^, ^29^, ^33^ ^]^ We chose borosilicate bioactive glass here because it is sufficiently supportive and safe compared to other materials such as PDMS and PU. Borosilicate bioactive glass provides sufficient mechanical support to maintain the structural integrity of the device, especially under varying ultrasound conditions. It also offers excellent biocompatibility and safety, reducing the risk of adverse reactions when used in biomedical applications. Moreover, borosilicate materials offer the advantages of controllable degradation and excellent bonding with both soft and hard tissues, making them conducive to long‐term implantation therapy.^[^ ^34^, ^35^ ^]^ Upon receiving external ultrasonic excitation, UP‐MPH‐TTD generates alternating electric fields with low intensity (1–3 V cm^−1^) and medium frequency (100–300 kHz). These electric fields act on the tubulin of proliferating cancer cells, interfering with the mitosis process, thereby inducing tumor cell apoptosis and inhibiting tumor growth (Figure 1c).^[^ ^36^, ^37^ ^]^ In preclinical cancer models, TTF demonstrates selective toxicity to proliferating cells through anti‐mitotic mechanisms, while having no effect on normal cells in non‐proliferative regions, such as the brain.^[^ ^20^, ^38^ ^]^

**Figure 2:**
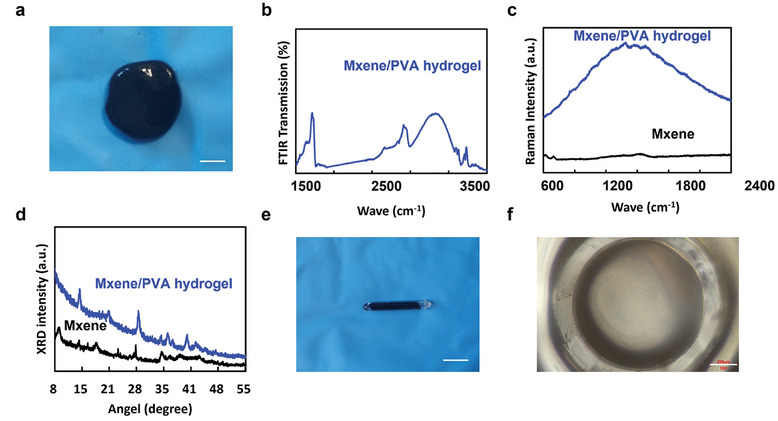
Characterization of the MXene/PVA hydrogel. a) Photo of the Mxene/PVA hydrogel. Scale bar: 3 mm. b–d) FTIR, Raman spectra, and XRD patterns of the MXene/PVA hydrogel. e) Photo of the UP‐MPH‐TTD. Scale bar: 3 mm. f) Cross‐sectional microstructure of borosilicate glass shell. Scale bar: 200 µm.

We mixed MXene nanosheets with a commercial PVA hydrogel “crystal clay” containing PVA, water, and anti‐dehydration additives, resulting in a stretchable MXene/PVA hydrogel composite (M‐gel).^[^ ^22^, ^39^, ^40^ ^]^ The prepared M‐gel is depicted in **Figure** **2**a with a strong hydrogen bonding peak at the wave crest of 3000–3800 cm^−1^ (Figure 2b). The structural composition of M‐gel prepared aligns with prior studies with similar Raman spectra of MXene nanosheets and M‐gel (Figure 2c).^[^ ^30^ ^]^ The powder X‐ray diffraction (XRD) pattern of MXene nanosheets showed prominent peaks corresponding to the (002), (006), and (008) planes, while the XRD of MXene/PVA hydrogels exhibited changes in the crystal structure (Figure 2d). The whole system is integrated in a borosilicate bio‐glass tube with a length of ≈6 mm (Figure 2e) an outer diameter of ≈1.2 mm, and an inner diameter of ≈0.9 mm (Figure 2f). The total volume of the device is less than 6.8 mm^3^. M‐gel is the core of the ultrasonic transducer system, converting mechanical energy into electrical energy by providing triboelectric output under periodic compression forces via the SVP model.^[^ ^29^, ^31^ ^]^ The ultrasonic wave disrupts the electrical equilibrium state generated by the friction between the water and the MXene nanosheet by compressing the microchannel on the surface of the MXene nanosheet, thus generating an electric field. As the total amount of charge within the M‐gel increases, the charge eventually induced on the device's surface also increases. Therefore, the electric field intensity can be adjusted by varying the M‐gel content.

### 2.2 UP‐MPH‐TTD for the GBM Treatment

**Figure 3:**
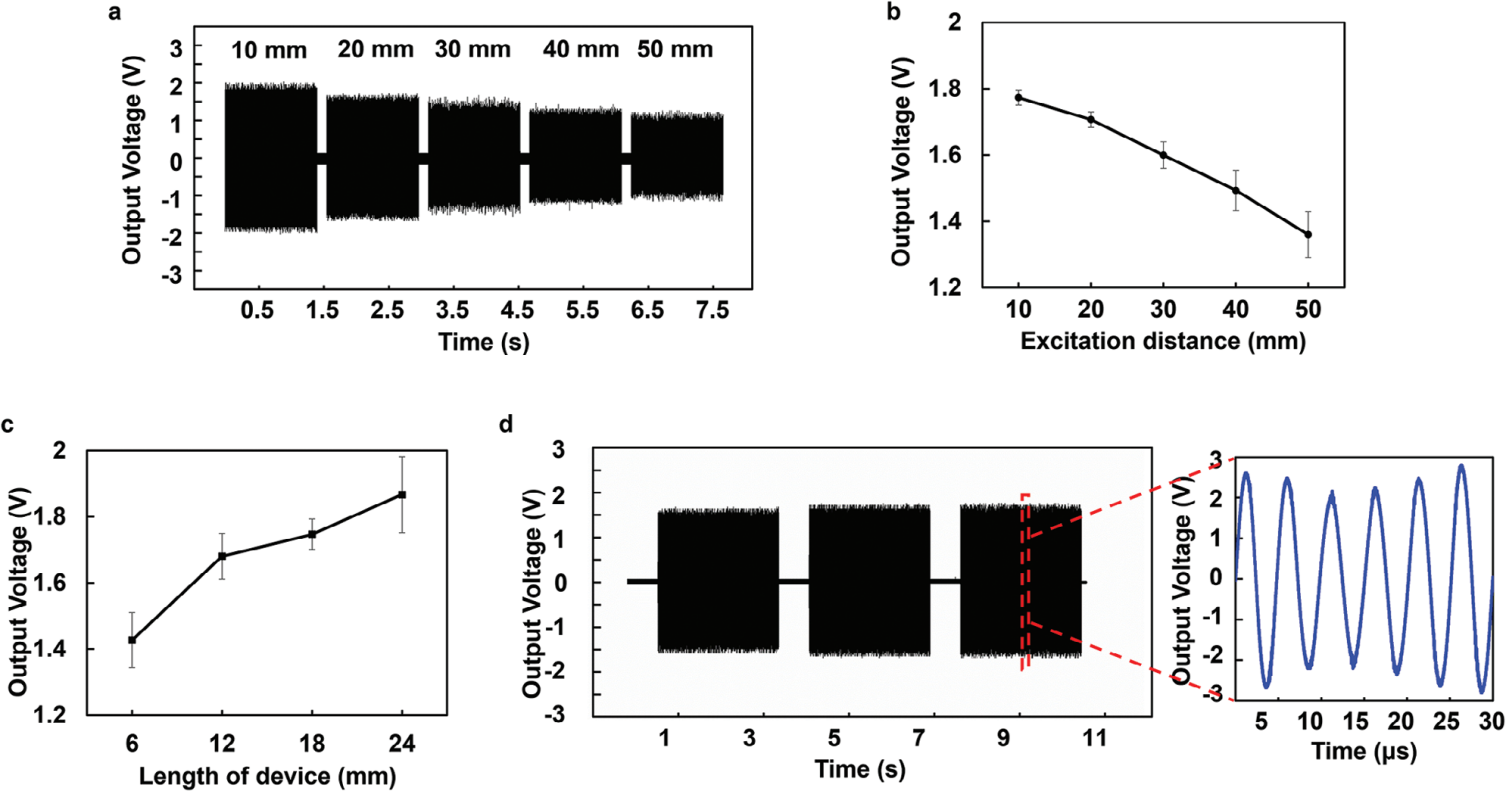
Output performance of the UP‐MPH‐TTDs. a,b) The output intensity of the UP‐MPH‐TTDs decreases as the excitation distance increases. c) Output voltages of the UP‐MPH‐TTDs with different lengths. d) Output of the UP‐MPH‐TTDs with a 0.3 W cm^−2^ ultrasonic excitation at 40 mm. The graphical insert explains the voltage waveform produced by the UP‐MPH‐TTDs at the microsecond time scale.

The therapeutic effect of TTF depends on two key parameters: the intensity and frequency of the electric field. Studies have shown that for GBM, a frequency of 200 kHz is the most effective.^[^ ^20^, ^22^, ^29^ ^]^ To obtain an electric field output that meets the optimal parameters for GBM treatment, the device output needs to be modulated. We adopt a straightforward method to tune the intensity and frequency of the electric field, avoiding the addition of external circuits that would increase the device's volume and energy consumption. The output field intensity can be adjusted by changing the ultrasonic power, the distance between the device and the ultrasonic wave source, or the device volume. The output frequency can be adjusted by changing the frequency of the ultrasonic wave source. According to calorimetric calculations, the power range of the ultrasound used in this work is between 0.3 and 0.4 W cm^−^^2^, which is a safe level for medical applications (100 µW cm^−2^ to a few mW/cm^2^).^[^ ^41^, ^42^, ^43^ ^]^ As shown in **Figure** **3**a,b, the distance between the ultrasonic wave source and the device is negatively correlated with the output intensity of the device (length = 6 mm, radius = 0.6 mm), allowing us to regulate the output voltage by adjusting the distance. Additionally, changing the device's structure can also alter the output strength, which increases with the length (Figure 3c) and the diameter (Figure S1, Supporting Information) of the device. To facilitate device implantation, we selected a diameter of 1.2 mm, balancing ease of implantation with sufficient output amplitude. Furthermore, the output frequency can also be modulated by altering the frequency of the ultrasonic source (Figure S2, Supporting Information). Subsequently, we found that changing the angle of the ultrasound source had minimal impact on the device's electric field output (Figure S3, Supporting Information). This indicates that the orientation of the device during implantation is not expected to significantly affect the therapeutic efficacy. The 6 mm UP‐MPH‐TTD treatment device was set to generate a sine‐wave alternating electric field with an intensity of 1–3 V cm^−1^ and a frequency of 200 kHz (Figure 3d), which is consistent with previously recommended treatment parameters. The stability of the output of the equipment was further proved in Figure S4 (Supporting Information), which indicated a high stability of device output for 700 s.

### 2.3 In Vitro and In Vivo Experiments

**Figure 4:**
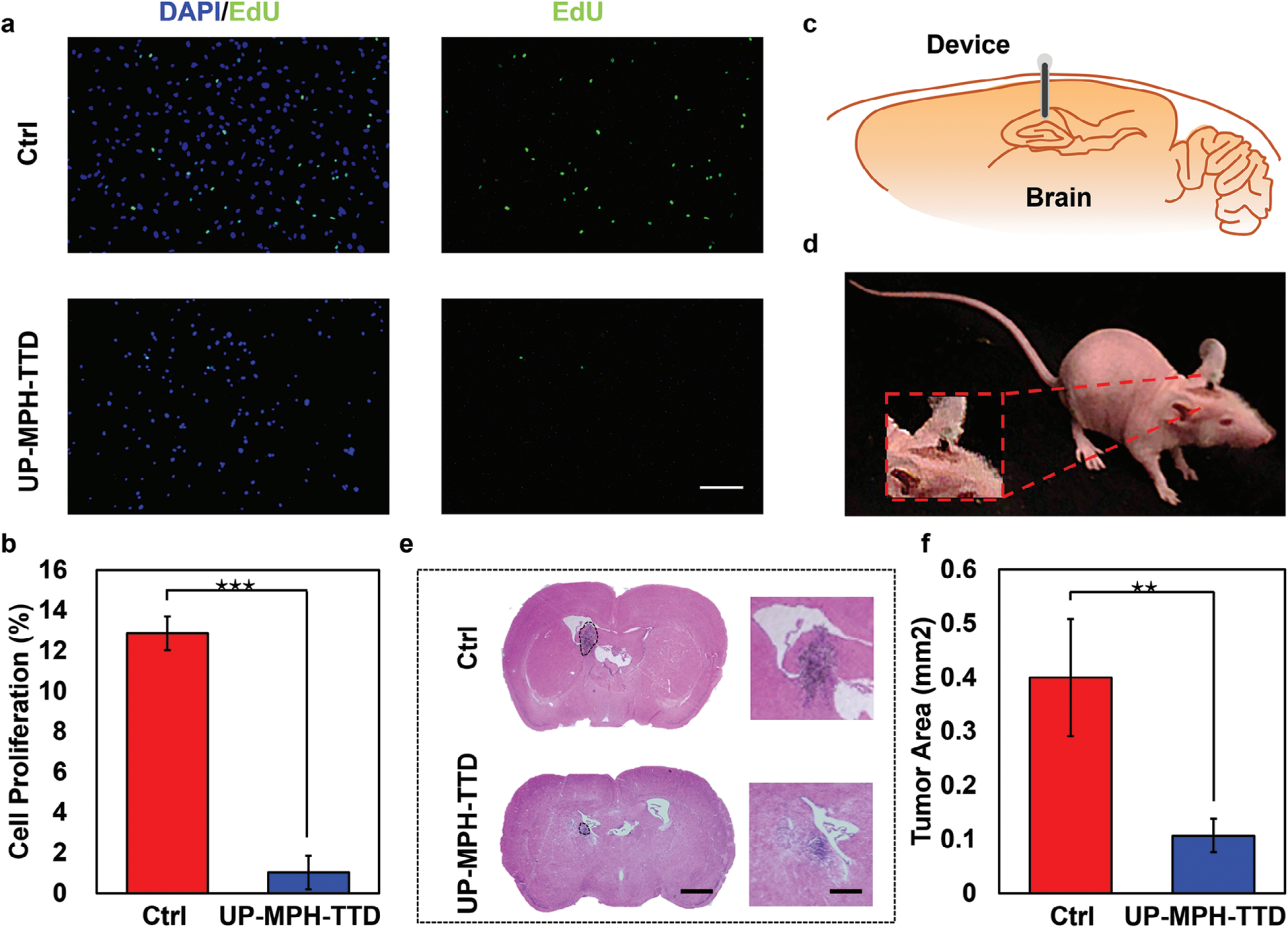
In vitro and in vivo experiments. a) Cell proliferation analysis by EdU assay. Green: EdU, Blue: Hoechst. Scale bar: 200 µm. b) Schematic diagram of the location of a device implanted in the mouse brain. c) Photograph of a mouse implanted with an UP‐MPH‐TTD. (d) Histological analysis of brain tumor shows that treated group developed much smaller tumors in the mouse brains compared to the control. Scale bar: 1 mm (Left) and 400 µm (Right). e) Statistical result of cell proliferation after 12‐h treatment. ^***^: *p*‐value < 0.001. f) Statistic results of tumor area between UP‐MPH‐TTD and control group. ^**^: *p*‐value < 0.01.

We divided the validation into two parts: in vivo and in vitro, to characterize the actual therapeutic effect of UP‐MPH‐TTD on GBM. First, GBM cells from clinical patients were characterized in vitro, and the electric field generated by UP‐MPH‐TTD under ultrasonic excitation was applied to both sides of the cultured cells through parallel plate electrodes. The electric field frequency was 200 kHz, and the field intensity was 1.4 to 1.6 V cm^−1^. After GBM cells were treated with the electric field for 12 h, the growth rates in the treated and control groups were evaluated and quantified using the 5‐ethynyl‐2′‐deoxyuridine (EdU) Cell Proliferation Reagent (**Figure** **4**a).

To further characterize the function of the device in vivo, the device was implanted into the brains of tumor‐bearing mice. The encapsulation of borosilicate bio‐glass provides sufficient rigidity for device implantation and prevents the hydrogel from expanding due to water absorption. GBM cells from clinical patients were implanted into the brains of male immunodeficient BALB/c nude mice to construct a mouse model of GBM, which was used to evaluate the therapeutic effect of the device in vivo. All models were constructed using the same batch of mice, with controls for consistency in experimental conditions and cell numbers to ensure similar tumor growth states. Subsequently, the UP‐MPH‐TTD device was implanted in the striatum of the mouse brain (Figure 4b,c) and stimulated by ultrasound (0.3–0.4 W cm^−2^). Histological analysis of the brain tissue was performed after 4 weeks of continuous treatment (20 h per day). As shown in Figure 4d, brain tumors developed in the brains of mice in the treatment group were much smaller than those in the control group.

**Figure 5:**
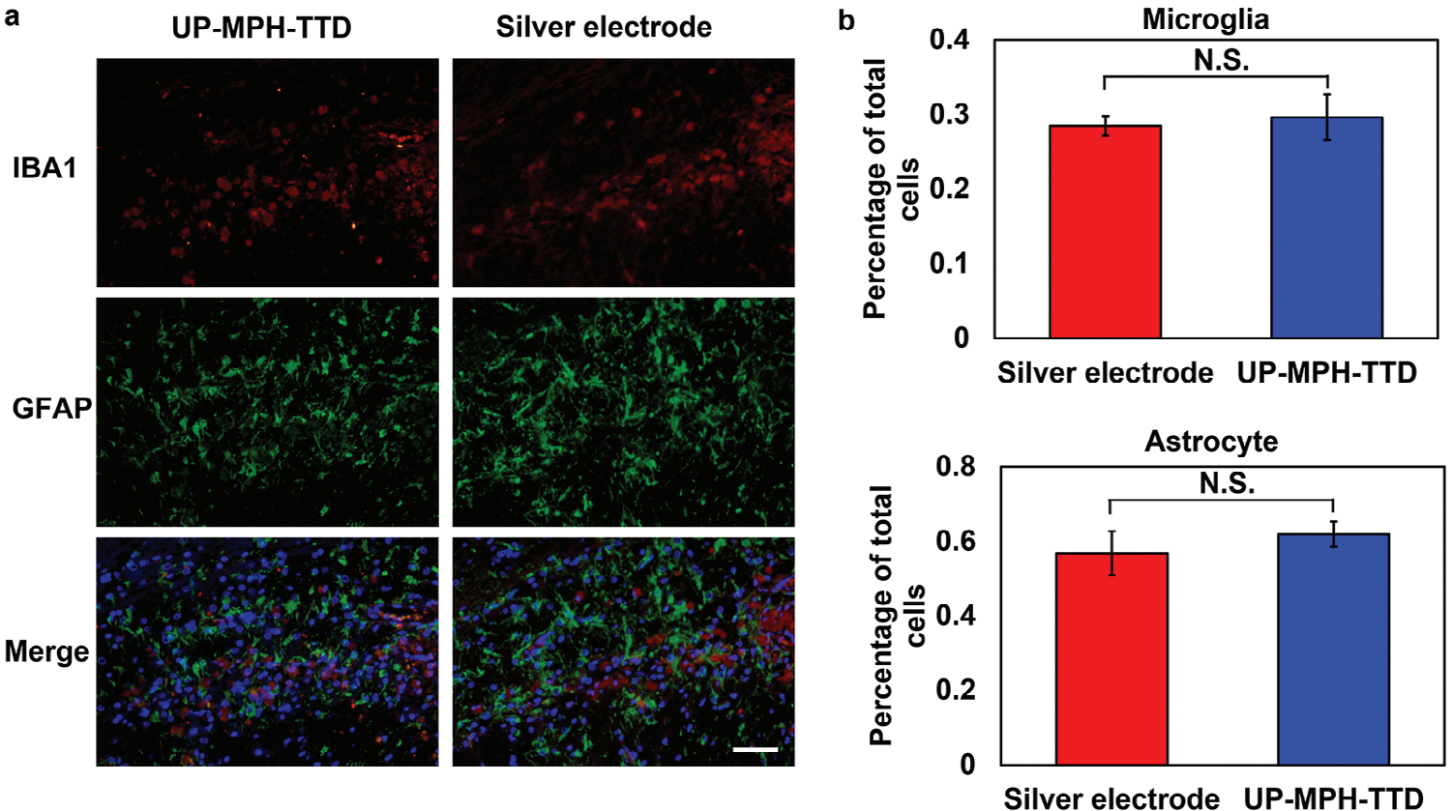
Biocompatibility evaluation of the UP‐MPH‐TTD and silver electrode. a) Immunostaining images of the brain slices: microglia cells (red) and astrocyte cells (green). Scale bar: 50 µm. b) Statistical analysis of microglia and astrocyte fraction of the UP‐MPH‐TTD groups and silver electrode groups. N.S.: not significant.

Quantitative results showed that the proliferation rate of GBM cells in vitro was significantly inhibited (*n* = 3, *p* < 0.001, *t*‐test), with an inhibition rate as high as 92% (Figure 4e), indicating that the device can effectively inhibit the growth of GBM cells in vitro. Statistical analysis showed that the tumor area of mice in the treatment group was 73% smaller than that of mice in the control group (*n* = 4, *p* < 0.01, *t*‐test), indicating a significant inhibiting effect in the treatment of GBM in vivo (Figure 4f). Compared with traditional treatment methods, our UP‐MPH‐TTD device demonstrated better efficacy than TMZ drug treatment alone (tumor growth inhibition value of 19.4%, *n* = 3, *p* < 0.001, *t*‐test) or TTF alone (tumor inhibition rate of 58%, *n* = 3, *p* < 0.05, *t*‐test), showing a significantly enhanced tumor inhibitory effect (Figure 5S).

As an implantable device, in addition to evaluating its feasibility in the treatment of GBM, its safety factors should also be considered. The device was implanted in animals for over one month to test the safety of the device. We did not observe any abnormal behaviors or affected survival rate with the device implanted. Reactive tissue response to our UP‐MPH‐TTD was assessed using immunohistochemical analysis of the peripheral brain tissue of SD rats implanted with UP‐MPH‐TTDs and conventional silver electrodes for more than a month. We assessed astrocyte and microglia responses to the device with glial fibrillary acidic protein (GFAP) and ionized calcium‐binding adaptor molecule 1 (Iba1). The immunostaining of activated astrocytes and microglia are shown in **Figure** **5**a. Notably, the inflammatory response caused by our device was comparable to that of conventional silver electrodes (*n* = 3, p > 0.05, *t*‐test), indicating that our device has similar biocompatibility to conventional electrodes and can achieve long‐term implantation therapy (Figure 5b).

## 3 Conclusion

In summary, we propose an implantable wireless UP‐MPH‐TTD that can be used to improve the prognosis of glioblastoma and treat brain tumors. The MXene/PVA hydrogel is a promising ultrasonic transducer material capable of generating sufficient electricity from ultrasound to produce TTF or power medical electronic devices. Wireless energy transmission through ultrasound not only overcomes the difficulties of traditional wiring methods but also provides a relatively safe energy transmission scheme. Device output modulation experiments show that the output intensity and frequency of UP‐MPH‐TTD are easily adjustable. Intensity modulation can be achieved by controlling the distance between the device and the ultrasonic source or by changing the device volume, allowing for customized GBM therapy. Moreover, the implantable design enables the device to accurately target brain tumors, greatly improving treatment efficiency. Particularly for multiple brain tumors, the location and quantity of UP‐MPH‐TTD can be flexibly adjusted according to the tumor sites. As an implantable device, UP‐MPH‐TTD is encapsulated in biocompatible borosilicate bioactive glass, which provides sufficient rigidity for easy implantation and good bonding with both hard and soft tissues. The results of immunohistochemical analysis further demonstrate the device's potential as a long‐term implant. Our device demonstrates greater precision and efficacy compared to traditional chemotherapy and electric field treatments. For patient T‐36 in our experiment, the inhibition rate of existing glioblastoma drug treatments is ≈19.4%, while TTF treatment achieves ≈58% inhibition of glioblastoma growth. In contrast, our UP‐MPH‐TTD device achieved a tumor inhibition rate of 73%, representing an improvement of ≈276% over single TMZ drug treatment and 26% over TTF treatment. These results collectively affirm that the UP‐MPH‐TTD device not only offers a powerful tool for inhibiting glioblastoma growth (inhibition rate of 92% in vitro and 73% reduction in tumor area in vivo) but also ensures safety for clinical applications. We conducted a thorough safety assessment by implanting the device in rats for over a month. During this period, we observed no abnormal behaviors or adverse effects on the survival rates of the animals, confirming the device's biocompatibility and safety for long‐term use. The device offers new hope for improving the prognosis of patients with brain tumors, particularly glioblastoma.

## 4 Experimental Section

### Synthesis and Characterization of MXene/PVA Hydrogel

The MXene‐based PVA hydrogel (MXene/PVA hydrogel) was prepared using the following process. An 8 wt% PVA solution was prepared by dissolving PVA 1788 powder (Shanghai Aladding Biochemical Technology Co., Ltd.) in ultrapure water with magnetic stirring at 90 °C until completely dissolved. Meanwhile, a 4 wt% sodium tetraborate solution was obtained by dissolving sodium tetraborate (Shanghai Macklin Biochemical Co., Ltd.) in ultrapure water at 50 °C, shaking the solution until completely dissolved. Then, the MXene nanosheets were added to the sodium tetraborate solution and subjected to ultrasonic treatment for 1 h to ensure even dispersion of the MXene nanosheets (thickness 100–200 nm, purity 54–68 wt%, Jiangsu XFNANO Materials Tech Co., Ltd.) in the solution. Finally, the two solutions were mixed to obtain the MXene/PVA hydrogel, with a MXene weight percentage of 4 wt%. The composition and phase structure of the prepared hydrogel were determined using Fourier Transform Infrared (FTIR) spectroscopy (V70, Germany), Raman spectroscopy (RENISHAW, Britain), and X‐ray diffraction (EMPYREAN, Holland).

### Fabrication and Characterization of the UP‐MPH‐TTD

The UP‐MPH‐TTD is a cylindrical structure with MXene/PVA hydrogel encapsulated inside a glass capillary (diameter: ≈1.2 mm) composed of borosilicate active glass. One end of the glass capillary is closed. Then, the prepared MXene/PVA hydrogel is inserted into the capillary, and a conductive copper wire is attached as an external electrode. The other end of the capillary is sealed with hot melt adhesive to prevent the MXene/PVA hydrogel from losing water. The length of the UP‐MPH‐TTD is controllable. The electrical outputs generated by the UP‐MPH‐TTD were measured and recorded using an oscilloscope (Rigol, DS2202) with a voltage probe (Rigol, RP3300) having a 1‐megohm input impedance. Ultrasound was generated and adjusted using a commercial ultrasonic transducer (THD‐M1) and generator (KM‐102). In most cases, two parallel plates were used to generate an electric field. The distance separating the two plates was carefully controlled to establish a nearly uniform electric field between them. The field intensity (E) was calculated by dividing the potential difference under working load (U) by the separating distance (d) between the plates. The calorimetric method was used to verify the excitation ultrasound energy for the UP‐MPH‐TTD. To verify the reproducibility of the hydrogel synthesis and device parameter tuning, 3 UP‐MPH‐TTDs for each condition were prepared. Output frequency was characterized using ultrasonic wave sources of 23, 25, 40, and 200 kHz, with a fixed ultrasonic power of 0.3–0.4 W cm^−^^2^. At 200 kHz, the device output amplitude was 1.56 V (standard deviation, 0.04) and the frequency was 205 kHz (standard deviation, 13.58). Devices with different diameters (2200 µm outer, 1863 µm inner; 1200 µm outer, 947 µm inner) and lengths (6, 12, 18, and 24 mm) showed output amplitudes of 1.56 V (standard deviation, 0.02) and 1.77 V (standard deviation, 0.13), and 1.43 V (standard deviation, 0.08) to 1.87 V (standard deviation, 0.11), respectively. Distance from the ultrasonic source (10–50 mm) also affected output, ranging from 1.77 V (standard deviation, 0.02) to 1.36 V (standard deviation, 0.07). Final device parameters were 6 mm in length, 0.6 mm in radius, with excitation parameters of 0.3–0.4 W cm^−^^2^ and 200 kHz frequency.

### Cell Culture

All the research procedures were approved by the IEC for clinical research and animal trials of the First Affiliated Hospital of Sun Yat‐sen University (2021723), and informed consent was obtained from all patients. Human clinical glioma tumors were surgically resected and collected for use. Tumor and surrounding cells were processed and separated through mechanical trituration, trypsinization, filtration, and centrifugation. Cells were cultured in Dulbecco's Modified Eagle Medium/Nutrient Mixture F‐12 (Hyclone, Logan, UT, USA) containing 10% fetal bovine serum, 2.5 mmol L^−1^ L‐glutamine, and 100 U mL^−1^ penicillin‐streptomycin solutions (Gibco, Grand Island, NY, USA). Cells were incubated at 37 °C in a humidified atmosphere with 5% CO2, with medium changed every other day.

### In Vitro Experiment

Clinical tumor cells were used to evaluate the effect of UP‐MPH‐TTD on cell proliferation using the EdU cell proliferation assay (BeyoClick EdU Cell Proliferation Kit with Alexa Fluor 488, Beyotime, Shanghai, China). Briefly, cells seeded on coverslips were cultured in DMEM/F12 medium and placed between parallel plates separated by a distance of 10 mm. One of the plates was grounded, and the other was connected to the generator. The electric field generated by the generator was measured in the medium as 1.4 to 1.6 V cm^−1^ at 200 kHz. Simultaneously, cells were incubated with 10 µm EdU for 12 h, followed by fixation with 4% paraformaldehyde (PFA) for 15 min and permeabilization with 0.3% Triton X‐100 for another 15 min at room temperature (RT). Subsequently, the samples were incubated with the Click Reaction Mixture at RT for 30 min in the dark and then incubated with Hoechst 33342 for 10 min. Images were captured using a fluorescence microscope (Cnoptec, Chongqing, China).

### In Vivo Experiment

All procedures involving animals were approved by the Animal Ethical Committee of Sun Yat‐sen University (SYSU‐IACUC‐2021‐B0989). The immunodeficient BALB/c nude mice were purchased and housed in Sun Yat‐sen University Experimental Animal Center under a 12‐h light/dark cycle and had ad libitum access to food and water. Eight BALB/c nude mice were randomly divided into two groups. All of them were injected with clinical tumor cells (T‐36) in the striatum (bregma, AP: 3.0 mm, ML: ±2.0 mm, DV: −2.5 mm) to construct an orthotopic transplantation tumor model. Mice were anesthetized with 5% chloral hydrate (intraperitoneal, 0.08 mL/10 g) to relieve pain during the surgical procedure. First, the surgical area of the mouse head was sterilized with a cotton ball containing 75% medical alcohol, and then the mouse's scalp was cut. A 1.3 mm diameter hole in the skull was drilled. Clinical gliomas cells (5 × 105 cells in 5 µL) were implanted into the right hemisphere perpendicularly at a location 1.5 mm posterior to the bregma, 1.5 mm lateral to the sagittal suture, and at a 3.5 mm depth from the skull surface. After the needle was removed, a Mxene/PVA hydrogel‐based generator was implanted into the mouse's brain at a depth of 3 mm along the needle track to generate an electrical field. At the same time, one burr hole was drilled for screws around the insertion site of the generator. Then, the generator was firmly fixed by the surrounding screw and dental cement. After suturing the skin over the mouse's head, they were individually placed in a standard rearing cage with ultrasound transducers 2 to 4 cm above. The ultrasound transducers were customized as larger as the cage, which could provide consistent ultrasound patterns in all areas. The UP‐TTD groups were treated with 200‐kHz TTFs at an intensity of 1.4 to 1.6 V cm^−1^ for 4 weeks. After 4 weeks of treatments, mice were anesthetized with chloral hydrate and perfused with normal saline and following 4% PFA. At the end of perfusion, brain tissues were removed by craniotomy and fixed in 4% PFA for histological sections. Before HE staining, the entire brains of the mice were sliced along the coronal plane at regular intervals (40 µm) to ensure that the tumor in the brain was completely sectioned and made into brain slices. Then, the brain slice with the largest tumor area was selected as the tumor area size of each mouse to ensure the scientific validity of the results.

### Biocompatibility Evaluation of the UP‐MPH‐TTD

All procedures involving animals were approved by the Animal Ethical Committee of Sun Yat‐sen University. SD rats, weighing 160 ± 5 g, were purchased and housed in Sun Yat‐sen University Experiment Animal Center. A UP‐MPH‐TTD and a traditional silver electrode were implanted into the brain of the mice respectively for further physiological evaluations. The depth of implantation was 3 mm. After 5 weeks of implantation, mice were anesthetized with chloral hydrate and perfused with normal saline and following 4% PFA. At the end of perfusion, brain tissues were removed by craniotomy and fixed in 4% PFA overnight. Coronal brain slices in the same depth range were selected for further analysis. The slices were pre‐treated using heat mediated antigen retrieval with sodium citrate buffer, permeabilized in 0.25% Triton X‐100 for 15 min, and then blocked with 4% BSA for 2 h room temperature or overnight at 4 °C. Then, samples were incubated with primary antibodies for 4 h at room temperature followed with secondary antibodies for 2 h. Images were captured with a fluorescence microscope (Cnoptec, Chongqing, China). The primary antibodies used were: GFAP (ab4648, Abcam, Cambridge, UK), IBA1 (ab178846, Abcam, Cambridge, UK). The secondary antibodies used were: Anti‐rabbit IgG (H + L) (Alexa Fluor 594 Conjugate, 8889, Cell Signaling Technology, Boston, MA, USA), anti‐mouse IgG (H + L) (Alexa Fluor 488 Conjugate, 4408, Cell Signaling Technology, Boston, MA, USA).

### Statistical Analysis

The bar and curve graph data were presented as the mean ± standard error of the mean (s.e.m.). The statistical data were analyzed with a one‐way analysis of variance (ANOVA) using SPSS statistics 25 (IBM SPSS Inc., NY, USA) and examined by student's *t*‐test at a 95% of confidence interval.

### Information of Clinical Cell Lines

Cells for all the tests were from clinical glioma patient with detailed information as follows:

• T‐36;
WHO grade: IV;
Histology: Glioblastoma;
Molecular background: IDH1(‐); TERT promoter region mutation; MGMT gene promoter methylation(+); BRCA2, CDKN2A, CDKN2B, PTEN deletion; CDK6, SMO amplification, PIK3CAp.H1047Y, STAG2p.E470 cancer driver gene mutation;
Prior treatment with radiation: There was no radiotherapy before the tumor was surgically removed.

## Conflict of Interest

The authors declare no conflict of interest.

## Author Contributions

X.H. and Z.Q. contributed equally to this work. B.X. designed and supervised the project, decided research methods and wrote the paper; J.X. provided clinical guidance; X.H. performed experiments and collected data; Z.Q. helped on animal experiments and wrote the paper; Y.Y., T.X., X.C., provided experiment supports. Competing interests: The authors declare that they have no competing interests. All data needed to evaluate the conclusions in the paper are present in the paper and/or the Supporting Information.

## Supporting information



Supporting Information

## Data Availability

The data that support the findings of this study are available from the corresponding author upon reasonable request.
