# Supplementary material for: Implantable Ultrasound‐Powered MXene/PVA Hydrogel‐Based Generator for Treatment of Glioblastoma
Source: Adv Sci (Weinh). 2024 Dec 12;12(5):2309610. doi: 10.1002/advs.202309610 (PMC11791951; doi:10.1002/advs.202309610)
Supplement: Supplementary file 1 — Supporting Information [file ADVS-12-2309610-s001.docx]

Supporting Information

**Figure S1. The output intensity of the UP-MPH-TTDs with different diameters.** Detection conditions: device length (6 mm), angle between device and ultrasonic source (0 degree), distance between device and ultrasonic source (40 mm), wave source power (0.3 W/cm²), wave source frequency (200 kHz).

**Figure S2. The output frequency of the device under excitation of ultrasonic wave sources with different frequency (23kHz, 28kHz, 40kHz, 200kHz).** Detection conditions: device diameter (1.2 mm), device length (6 mm), angle between device and ultrasonic source (0 degree), distance between device and ultrasonic source (40 mm), wave source power (0.3 W/cm²).

**Figure S3. The output intensity of the UP-MPH-TTDs with different angles between UP-MPH-TTDs and ultrasonic source.** Detection conditions: device diameter (1.2 mm), device length (6 mm), distance between device and ultrasonic source (40 mm), wave source power (0.3 W/cm²), wave source frequency (200 kHz).

**Figure S4. The voltage waveform lasting 700s generated by UP-MPH-TTD.** Detection conditions: device diameter (1.2 mm), device length (6 mm), angle between device and ultrasonic source (0 degree), distance between device and ultrasonic source (40 mm), wave source power (0.3 W/cm²), wave source frequency (200 kHz).

**Figure S5. The tumor growth inhibition value of UP-MPH-TTD, TMZ and TTF.** The tumor growth inhibition value of UP-MPH-TTD (73%) was significantly higher than that of TMZ (19.4%) and TTF (58%). *: p-value < 0.05; ***; p-value < 0.001.
